# Alcohol Consumption in Relation to Risk and Severity of Chronic Widespread Pain: Results From a UK Population‐Based Study

**DOI:** 10.1002/acr.22604

**Published:** 2015-08-26

**Authors:** Gary J. Macfarlane, Marcus Beasley, Gordon J. Prescott, Paul McNamee, Philip C. Hannaford, John McBeth, Karina Lovell, Phil Keeley, Deborah P. M. Symmons, Steve Woby, John Norrie

**Affiliations:** ^1^ Gary J. Macfarlane, PhD, MD(Hons), Marcus Beasley, BSc, MSc: School of Medicine and DentistryUniversity of AberdeenAberdeenUK

## Abstract

**Objective:**

To determine whether the reported level of alcohol consumption is associated with the likelihood of reporting chronic widespread pain (CWP) and, among persons with CWP, the associated disability.

**Methods:**

In a population‐based study in 2 areas of the UK, participants self‐completed a postal questionnaire. They were classified according to whether they met the American College of Rheumatology definition of CWP and whether the pain was disabling (Chronic Pain Grade III or IV). They reported their usual level of alcohol consumption. Potential confounding factors on which information was available included age, sex, cigarette smoking, employment status, self‐reported weight and height, and level of deprivation.

**Results:**

A total of 13,574 persons participated (mean age 55 years, 57% women) of whom 2,239 (16.5%) had CWP; 28% reported never regularly consuming alcohol, 28% reported consuming up to 5 units/week, 20% reported 6–10 units/week, and 24% reported >10 units/week. Among persons with CWP, disability was strongly linked to level of alcohol consumption. Prevalence of disability decreased with increasing alcohol consumption up to 35 units/week (odds ratio [OR]_21–35 units alcohol/week versus never drinkers_ 0.33 [95% confidence interval (95% CI) 0.19–0.58]) adjusted for confounders. A similar relationship was found between reporting CWP and level of alcohol consumption (adjusted OR_21–35 units alcohol/week versus never drinkers_ 0.76 [95% CI 0.61–0.94]).

**Conclusion:**

This study has demonstrated strong associations between level of alcohol consumption and both CWP and related disabilities. However, the available evidence does not allow us to conclude that the association is causal. The strength of the associations means that specific studies to examine this potential relationship are warranted.

## INTRODUCTION

The etiology of chronic widespread pain (CWP) is complex. Large‐scale, cross‐sectional population studies have provided insight into the characteristics of persons who report CWP; they report high levels of psychological distress, poor sleep quality and fatigue, and other somatic symptoms, and they demonstrate specific attitudes, beliefs, and behaviors about health 1. Fibromyalgia (the characteristic feature of CWP) has been linked to adverse life events 2 and specifically to maltreatment in childhood 3. However, some of these “associated” features may be consequences rather than precedents of the onset of CWP 4, and there is evidence that the relationship with potential etiologic factors, such as adverse childhood factors, may partly be explained by differential recall 5, 6.

Box 1 **Significance & Innovations**

- Clinic patients with fibromyalgia who consume moderate levels of alcohol have been shown, in a single US study, to have lower levels of disability.
- We have replicated this result in a population‐based study of persons with chronic widespread pain (CWP) in the UK.
- We have newly shown that the usual level of alcohol consumption was associated with the likelihood of whether a person reports CWP.
- Although we can't say that the association is causal at present, its magnitude makes a strong case for it to be the specific focus of future studies.

Cohort studies have allowed us to identify predictors of CWP onset, such as psychological distress, poor sleep quality, illness attitudes, and behavior 7, 8. However, since epidemiologic studies have demonstrated that the first onset of CWP is uncommon in mid‐adulthood, even such longitudinal studies are likely to be examining predictors of episode onset, and lifestyle factors, experiences, and attitudes may well have been affected by previous experience of CWP. More generally, establishing the role of lifestyle factors in the etiology of chronic diseases from observational studies is challenging, and there are several examples where erroneous conclusions have been drawn. For example, while observational studies suggested that higher levels of ß‐carotene were associated with reduced cardiovascular mortality, clinical trials of ß‐carotene supplementation demonstrated an increased risk 9. The likely reason for this was confounding factors, some unmeasured and others measured with error 10.

In this context it was recently reported that among clinic patients with fibromyalgia, moderate alcohol consumption (in comparison to abstaining) was associated with reduced symptom severity and increased quality of life 11. An accompanying editorial posed the question, “Can alcohol consumption be an alternative treatment for fibromyalgia?” 12. If this were to be considered, then the observed relationship between alcohol consumption and fibromyalgia severity and quality of life would need to be causal rather than just an association, and the direction of causality would need to be from alcohol consumption to symptom severity rather than vice versa.

The purpose of the current analysis, using a previously conducted epidemiologic study, was to determine whether we could confirm, among persons with CWP, that moderate alcohol consumption was associated with lower disability and, second, for the first time to examine whether usual level of alcohol consumption was associated with likelihood of reporting CWP. Finally, for any association observed we wanted to consider the likelihood that the association was causal.

## MATERIALS AND METHODS

The MUSICIAN (Managing Unexplained Symptoms [chronic widespread pain] In Primary Care: Involving Traditional and Accessible New Approaches) trial (see Appendix A for investigators) is a randomized controlled trial investigating the management of CWP. It tested the efficacy of telephone‐delivered cognitive–behavioral therapy and a community‐based exercise program in comparison to usual care. Since identification of eligible patients was difficult from primary care records, a large‐scale recruitment survey was undertaken, and it is data from this survey, the size of which was determined by power calculations of the trial, which are used in the current analysis. The study areas were the city of Aberdeen, Scotland and North Cheshire, England. The city of Aberdeen is an affluent city with high employment, particularly in the fields of oil and higher education. North Cheshire is a semirural area with market towns, but from where residents also commute to major industrial cities. The list of persons ages ≥25 years on participating general practice registers was used as the sampling frame for the recruitment survey. Since more than 95% of persons residing in the UK are registered with a general practitioner, this represents a suitable population sampling frame. A random sample of 45,949 persons on the sampling frame was sent a questionnaire by post, and nonresponders received a further questionnaire 2 weeks later. The survey was started in April 2008, and mailings were undertaken in 3 waves in order to accommodate treatment within the trial. The survey finished in February 2009. The study received ethics approval from the Cheshire Local Ethics Committee, UK (reference 07/Q1506/61).

#### Pain

The questionnaire asked, “Thinking back over the past month, have you had any aches or pains that have lasted for one day or longer?” Respondents answering positively were invited to shade the location(s) of their pain on 4‐view body manikins. From this we were able to identify persons who reported pain satisfying the definition of CWP used in the 1990 American College of Rheumatology (ACR) criteria for fibromyalgia 13. Respondents also completed the Chronic Pain Grade (CPG) scale, which measures impact from grade I (low disability/low intensity) to IV (high disability, severely limiting), and was developed in the US, but validated in the UK for use in postal questionnaires 14.

#### Alcohol consumption

Respondents were asked, “Have you ever drunk alcohol regularly (at least once per week) for a period of one month or longer?” If the response was yes, respondents were asked, “How many units of alcohol do you drink, on average, per week?” They were reminded that 1 unit of alcohol is a half‐pint of average strength beer/lager or 1 small glass of wine or 1 single measure of spirits.

#### Confounding factors

Information was available on respondent age, sex, employment status, usual number of cigarettes smoked, and self‐reported weight and height (allowing calculation of body mass index [BMI]). Data on deprivation were derived, based on a participant's home address postal code, using the Carstairs Index 15. This area‐based index uses measures of social class, car ownership, overcrowding, and unemployment to estimate deprivation. Postal code information was available for all participants in the North Cheshire primary care network, but was only available from those in the Aberdeen primary care network who consented to be contacted for further studies.

#### Statistical analysis

The outcomes used in logistic regression analyses were 1) in the analysis among persons reporting disabling CWP (severe chronic pain; CPG grades III and IV) and 2) in the analysis involving all subjects, those reporting CWP. The analysis was adjusted for the available potential confounding variables listed above. Secondary analyses were also done using those participants for whom Carstairs Index scores were available, first an analysis adjusted for the confounding factors in the main analysis, and then repeated, additionally adjusting for the Carstairs Index. The associations are reported as odds ratios (ORs) with 95% confidence intervals (95% CIs).

## RESULTS

A total of 13,574 persons responded and provided useable data for the current analysis. The mean age was 55 years (interquartile range [IQR] 43–67 years), 57% were women, and 16.5% (n = 2,239) satisfied the ACR definition of CWP (prevalence: Aberdeen 16.5%, Cheshire 16.6%; *P* = 0.87). With respect to alcohol consumption, 28% reported that they had never regularly drunk alcohol, 28% reported up to 5 units/week on average, 20% reported 6–10 units/week, 15% reported 11–20 units/week, and 7% reported 21–35 units/week, while 2% reported that they drunk on average >35 units/week. There were differences in alcohol consumption between the 2 study sites (*P* < 0.001): a greater proportion of persons in Aberdeen reported never having drunk regularly (Aberdeen 31.2% versus Cheshire 22.8%), while drinking >10 units/week was less common in Aberdeen (21.2% versus 29.6%).

Carstairs Index of deprivation scores were available for 8,983 persons. Those with Carstairs Index scores were younger than those without (median age 55 versus 56 years). Although significant (*P* < 0.5), the absolute difference was small. They were also significantly more likely to be ex‐smokers (30.8% versus 28.7%), to have drunk alcohol regularly (74.5% versus 67.4%), and to report CWP (18.7% versus 12.2%). There were no differences in sex, BMI, or employment status, and there were no differences among those with CWP in reported disability.

#### Is reported alcohol consumption related to disability among persons with CWP?

Among persons who reported CWP, disabling pain (CPG III or IV) was strongly related to reported alcohol consumption (Table 1). The proportion of persons who never drunk regularly and who had disabling pain was 47.2%, and this percentage decreased with increasing reported alcohol consumption, such that only 18.6% of persons with CWP who reported drinking 21–35 units/week had disabling pain, a difference that remained highly statistically significant after adjustment for confounders (adjusted OR 0.33 [95% CI 0.19–0.58]). Among persons drinking at higher levels (>35 units/week), the proportion with disabling pain was similar to never regular drinkers (51.1%; adjusted OR 0.78 [95% CI 0.37–1.68]). There was no statistically significant difference in the relationship between alcohol consumption and CWP disability in men and women, as assessed by an interaction term in the logistic regression model; however, the lowest disability occurred at 11–20 units/week in women (17.5% versus 47.6% among never drinkers: adjusted OR 0.35 [95% CI 0.21–0.60]) in comparison to 21–35 units/week in men (15.5% versus 45.5% among never drinkers: adjusted OR 0.27 [95% CI 0.12–0.61]). Similar patterns for the relationship between alcohol consumption and disability were observed among the group for whom Carstairs Index scores were available (Table 2). After adjustment for deprivation, the OR for reporting disabling pain in those drinking 21–35 units/week compared to those who never drunk regularly was 0.30 (95% CI 0.16–0.57).

**Table 1:** The relationship between reported alcohol consumption and pain disability among persons with CWP^a^

Reported units of alcohol per week, no.	CPG I/II		CPG III/IV		Crude OR (95% CI)	Adjusted OR (95% CI)
	No.	%	No.	%		
Never drunk regularly	352	52.9	314	47.2	1.00	1.00
0–5	396	67.1	194	32.9	0.55 (0.44–0.69)	0.65 (0.50–0.84)
6–10	282	75.4	92	24.6	0.37 (0.28–0.48)	0.49 (0.36–0.67)
11–20	216	82.1	47	17.9	0.24 (0.17–0.35)	0.36 (0.24–0.54)
21–35	96	81.4	22	18.6	0.26 (0.16–0.42)	0.33 (0.19–0.58)
>35	23	48.9	24	51.1	1.17 (0.65–2.11)	0.78 (0.37–1.68)
Total	1,365	66.3	693	33.7		

a CWP = chronic widespread pain; CPG = Chronic Pain Grade; OR = odds ratio; 95% CI = 95% confidence interval.

**Table 2:** The relationship between reported alcohol consumption and pain disability among persons with CWP with Carstairs Index scores available^a^

Reported units of alcohol per week, no.	CPG I/II		CPG III/IV		Crude OR (95% CI)	Adjusted OR (95% CI)	Adjusted for deprivation OR (95% CI)
	No.	%	No.	%			
Never drunk regularly	248	51.9	230	48.1	1.00	1.00	1.00
0–5	311	67.3	151	32.7	0.52 (0.40–0.68)	0.60 (0.45–0.81)	0.64 (0.47–0.89)
6–10	225	76.3	70	23.7	0.34 (0.24–0.46)	0.45 (0.31–0.64)	0.47 (0.33–0.67)
11–20	164	82.8	34	17.2	0.22 (0.15–0.38)	0.31 (0.20–0.50)	0.34 (0.21–0.55)
21–35	77	81.1	18	19.0	0.25 (0.15–0.43)	0.28 (0.15–0.52)	0.30 (0.16–0.57)
>35	18	48.7	19	51.4	1.14 (0.58–2.22)	0.66 (0.28–1.56)	0.66 (0.28–1.58)
Total	1043	66.7	522	33.4			

a CWP = chronic widespread pain; CPG = Chronic Pain Grade; OR = odds ratio; 95% CI = 95% confidence interval.

#### Is reported alcohol consumption related to the likelihood of having CWP?

Among persons who responded to the survey and reported never having drunk alcohol regularly, the prevalence of CWP was 19.8% (Table 3). The prevalence of CWP decreased with increasing alcohol consumption such that among persons who reported 11–20 units/week or 21–35 units/week the prevalence of CWP was 13.1%, a large and significant decrease compared to never regular drinkers, after adjustment for confounding factors (adjusted OR 0.75 [95% CI 0.64–0.88] and adjusted OR 0.76 [95% CI 0.61–0.94], respectively). Persons who reported drinking more than 35 units/week of alcohol had a prevalence similar to those who never drank alcohol regularly (20.2%; adjusted OR 0.90 [95% CI 0.64–1.27]). There was no statistically significant difference in the relationship between alcohol consumption and CWP prevalence in men and women, as assessed by an interaction term in the logistic regression model; however, the lowest prevalence in women was among those drinking 11–20 units/week (prevalence 14.8% versus 21.0% among never regular drinkers; prevalence adjusted OR 0.76 [95% CI 0.61–0.95]), while in men the lowest prevalence was among those drinking 21–25 units/week (prevalence 11.8% versus 16.7% among never regular drinkers; prevalence adjusted OR 0.68 [95% CI 0.51–0.91]). The same relationships were observed between alcohol consumption and prevalence of CWP in those with Carstairs Index scores available (Table 4). After additional adjustment for deprivation, the ORs for reporting CWP in the groups drinking 11–20 units/week and 21–35 units/week compared to those who had never drunk regularly were, respectively, 0.68 (95% CI 0.56–0.82) and 0.72 (95% CI 0.56–0.93).

**Table 3:** The relationship between reported alcohol consumption and CWP^a^

Reported units of alcohol per week, no.	CWP, no		CWP, yes		Crude OR (95% CI)	Adjusted OR (95% CI)
	No.	%	No.	%		
Never drunk regularly	3,039	80.2	752	19.8	1.00	1.00
0–5	3,225	83.6	634	16.4	0.79 (0.71–0.89)	0.89 (0.79–1.01)
6–10	2,247	84.7	405	15.3	0.73 (0.64–0.83)	0.88 (0.76–1.01)
11–20	1,811	86.9	274	13.1	0.61 (0.53–0.71)	0.75 (0.64–0.88)
21–35	807	86.9	122	13.1	0.61 (0.50–0.75)	0.76 (0.61–0.94)
>35	206	79.8	52	20.2	1.02 (0.74–1.40)	0.90 (0.64–1.27)
Total	11,335	83.5	2,239	16.5		

a CWP = chronic widespread pain; OR = odds ratio; 95% CI = 95% confidence interval.

**Table 4:** The relationship between reported alcohol consumption and CWP among those with Carstairs Index scores available^a^

Reported units of alcohol per week, no.	CWP, no		CWP, yes		Crude OR (95% CI)	Adjusted OR (95% CI)	Adjusted for deprivation OR (95% CI)
	No.	%	No.	%			
Never drunk regularly	1,772	77.2	522	22.8	1.00	1.00	1.00
0–5	2,107	81.0	495	19.0	0.80 (0.69–0.92)	0.90 (0.78–1.04)	0.91 (0.79–1.05)
6–10	1,452	81.9	320	18.1	0.75 (0.64–0.87)	0.90 (0.76–1.06)	0.91 (0.78–1.08)
11–20	1,262	86.0	205	14.0	0.55 (0.46–0.66)	0.67 (0.55–0.80)	0.68 (0.56–0.82)
21–35	574	85.5	97	14.5	0.58 (0.45–0.73)	0.70 (0.55–0.91)	0.72 (0.56–0.93)
>35	136	76.8	41	23.2	1.02 (0.71–1.47)	0.86 (0.58–1.29)	0.87 (0.58–1.30)
Total	7,303	81.3	1,680	18.7			

a CWP = chronic widespread pain; OR = odds ratio; 95% CI = 95% confidence interval.

## DISCUSSION

This large population‐based study has found that persons with CWP are less likely to report their symptoms as disabling if they also report regularly consuming alcohol, confirming a previous report in a smaller clinic study of patients with fibromyalgia. Disability decreases with increasing reported alcohol consumption, but at the very highest levels of alcohol consumption (>35 units/week) disability is similar to those who have never regularly consumed alcohol. We also report for the first time that level of alcohol consumption is also related to the likelihood of reporting CWP. Prevalence is highest in those who have never regularly drunk alcohol or those reporting very high consumption (>35 units/week). Among other drinkers the likelihood of reporting CWP decreases with increasing consumption.

There are a number of methodologic issues to consider in interpreting the results from this analysis. First, it is well known that there is bias in the self‐reporting of alcohol consumption in surveys 16. Respondents are likely to have underestimated their alcohol consumption; the effect of this underreporting, provided it is nondifferential between persons with and without CWP, is that it would cause us to underestimate an effect since, for example, persons with high consumption were erroneously classified as drinking lower amounts of alcohol. Comparison with national UK surveys of alcohol consumption is problematic since these have generally tried to estimate the amount of alcohol drunk over a defined time in units, whereas we asked subjects to classify themselves into ranges of average alcohol consumption. However, since the maximum recommended alcohol consumption for men in the UK is 21 units/week (>14 units/week for women), surveys generally report the percentage of men exceeding this amount, and we can compare this with the percentage in the current study reporting 21–35 and >35 units/week. The 2008 General Lifestyle Survey in the UK, which involved 16,407 persons completing a questionnaire (74% participation rate), reported that 16.7% of men drank more than 21 units/week 17, which compares with 16.6% of men reporting alcohol consumption in the top 2 categories of the current study. For women, 8.4% reported consuming more than 14 units/week, which compares with 13.9% reporting more than 10 units/week, and 2.8% reporting more than 20 units/week in the current study. It therefore suggests that our reported levels of alcohol consumption in the current study are consistent with national surveys in the UK. Second, the study was not designed with the intention of addressing the current hypotheses, and therefore we do not have all the data that we would have wished for, particularly with respect to potential confounding factors. Ideally we would have had lifestyle factors (e.g., exercise) and individual characteristics (e.g., depression), which are known to be markers of CWP onset and related to alcohol consumption. Nevertheless, the groups that have been shown to be at low risk of reporting CWP in this analysis (i.e., those with moderate‐high alcohol consumption) are those that are likely to have lifestyle factors and individual characteristics (e.g., low mood, low levels of exercise) that would have put them at high risk of onset. Therefore, it is not evident whether the ability to adjust for such factors would have attenuated or increased the associations demonstrated. However, it was of note in this study, that after adjustment for levels of deprivation (using the Carstairs Index) the observed associations between alcohol consumption and CWP and disability were largely unchanged.

We are only aware of a single study that has specifically focused on the topic of fibromyalgia (that has CWP as its characteristic feature) and alcohol consumption 11. We have confirmed their results, studying CWP, that alcohol consumption is associated with reduced disability from fibromyalgia. Adding weight to the confirmation is the fact that while the results were similar, the studies were conducted in different countries (US versus UK), used different sampling frames (fibromyalgia treatment program versus population study), defined the outcome differently (CWP versus fibromyalgia), and collected information on alcohol consumption in different ways. Further, we have additionally found that level of alcohol consumption is also related to the likelihood of reporting CWP and not just its severity. The current study is also considerably larger, including more than 13,000 participants, more than 2,000 of whom had CWP. The authors of the previous study postulated a number of mechanisms linking alcohol consumption with reduced disability, including that the effects were mediated through γ‐aminobutyric acid (GABA) production. Ethanol enhances GABA release in the multiple brain regions 18 and, although they noted that a recent study had shown decreased levels of GABA in the brains of persons with fibromyalgia, this was only observed in a single area, i.e., the anterior insula, while no differences were found in the posterior insula, occipital cortex, or anterior cingulate 19.

Aside from consideration of the possible mechanisms whereby an association is observed, we think it is important to evaluate to what extent a causal mechanism is likely. Hill 20, in proposing his factors to consider whether an association was causal or not, identified one necessary criterion: temporality. The postulated risk factor must precede the onset of disease (or symptoms). From the available evidence of the 2 studies, it is not possible to determine that this criterion is met. Both were cross‐sectional studies and could be observing the consequences of having CWP of a particular severity. For example, in our study it may be that persons who have less disabling symptoms have few restrictions on their daily activities (including social activities) and are able to more fully engage in pastimes that give rise to opportunities for social consumption of alcohol. That may be true also for the comparison of persons with and without CWP. However, in our current study we have a category of “never having regularly consumed alcohol,” and persons in this category have a high prevalence of CWP and (if they have CWP) high levels of disability. Provided that persons are truthfully answering the questionnaire, their alcohol consumption cannot have changed after the development of fibromyalgia and does suggest that persons who have never consumed alcohol have a high risk of symptoms/disabling symptoms. In terms of other factors proposed by Hill, only strength (i.e., the observed associations are large) and biologic gradient (i.e., risk appears to be related to dose) are definitely satisfied. In terms of specificity (i.e., is the relationship only observed with CWP/fibromyalgia?), it is of note that a relationship between alcohol consumption and health has been observed for both general health and specific health conditions 21. In a seminal prospective study of approximately half a million US adults followed from 1982–91, Thun et al 22 reported mortality rates from cardiovascular disease to be significantly lower among men (relative risk [RR] 0.7, 95% CI 0.7–0.8) and women (RR 0.6, 95% CI 0.6–0.7) reporting at least 1 drink daily (i.e., at least 7 units/week) than among nondrinkers, with little relation to the level of consumption. Overall mortality rates were lowest among those consuming 1 drink per day, although mortality rates increased among heavy drinkers. A so‐called J‐shaped pattern of alcohol consumption and overall mortality has been reported in other major studies 23, 24. Specifically, alcohol consumption has also been related to increased mood, quality of life 25, and physical health 26. Therefore, any effect of alcohol consumption on pain is not specific, and an alternative mechanism to a direct effect is that alcohol has positive effects on general health, which influences the reporting of pain. Indeed, general health status has been found to be a strong predictor of pain onset in epidemiologic studies 27, 28. Alternatively, alcohol may have no positive effects on health at all either directly or indirectly, and drinking within limits that are regarded in society as normal may simply be a marker or indicator for those with healthier lifestyles and beneficial levels of certain risk factors 21.

In summary, this study has demonstrated associations between alcohol consumption and likelihood of reporting CWP and related disability. However, the available evidence from 2 studies does not allow us to conclude that the association is causal. It should not be interpreted that alcohol has a therapeutic benefit, and we further note that, in these study populations, a significant proportion of persons already report consuming more than the recommended limit. The magnitude of the associations (which are as strong as any other identified associated factor with CWP) does mean that further investigation and specific studies to examine this potential relationship are warranted. If such an association were true, it is unlikely to be directly beneficial to management; it may, however, provide clues to the mechanisms of development and outcome for CWP and fibromyalgia.

## AUTHOR CONTRIBUTIONS

All authors were involved in drafting the article or revising it critically for important intellectual content, and all authors approved the final version to be submitted for publication. Prof. Macfarlane had full access to all of the data in the study and takes responsibility for the integrity of the data and the accuracy of the data analysis.

**Study conception and design.** Macfarlane.

**Acquisition of data.** Macfarlane, Beasley.

**Analysis and interpretation of data.** Macfarlane, Beasley.

## MUSICIAN STUDY INVESTIGATORS

Investigators on the MUSICIAN study were as follows: Gordon J. Prescott, Paul McNamee, Philip C. Hannaford (University of Aberdeen); John McBeth, Karina Lovell, Phil Keeley, Deborah P. M. Symmons (University of Manchester); Steve Woby (Penine Acute NHS Trust). John Norrie was originally an investigator of the MUSICIAN study while Director of the Centre for Health Care Randomised Trials at the University of Aberdeen.
